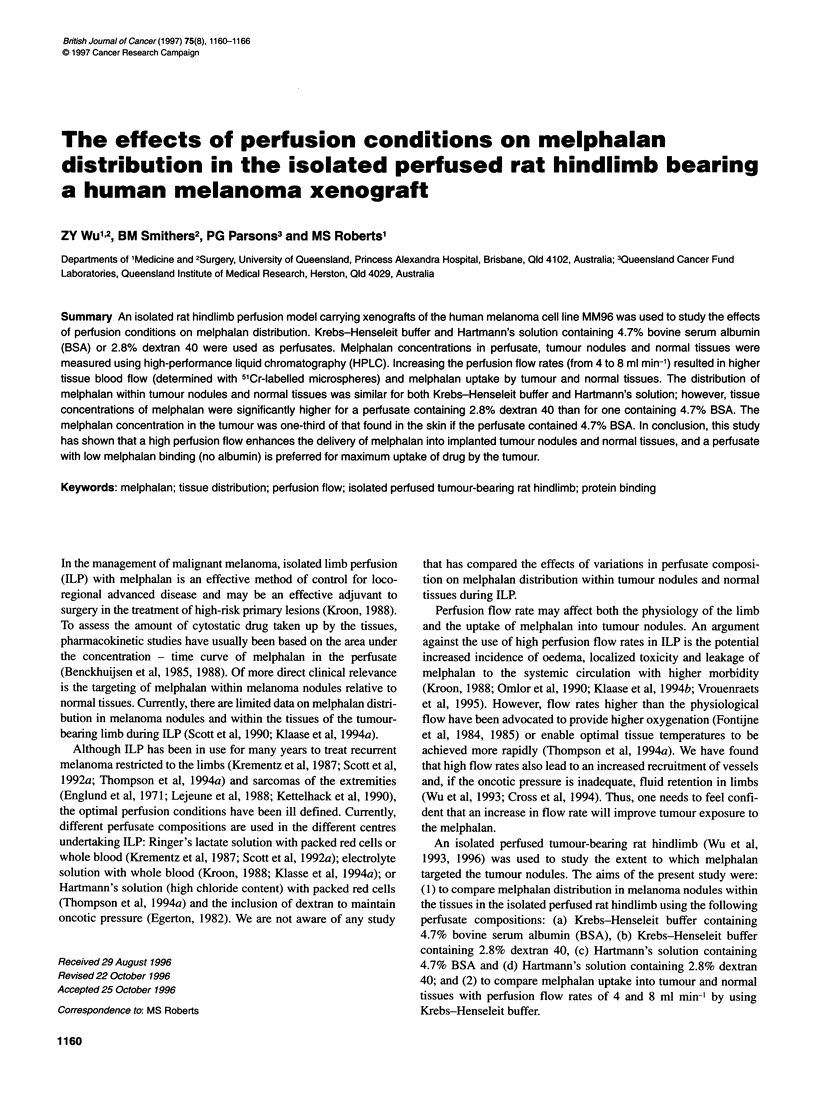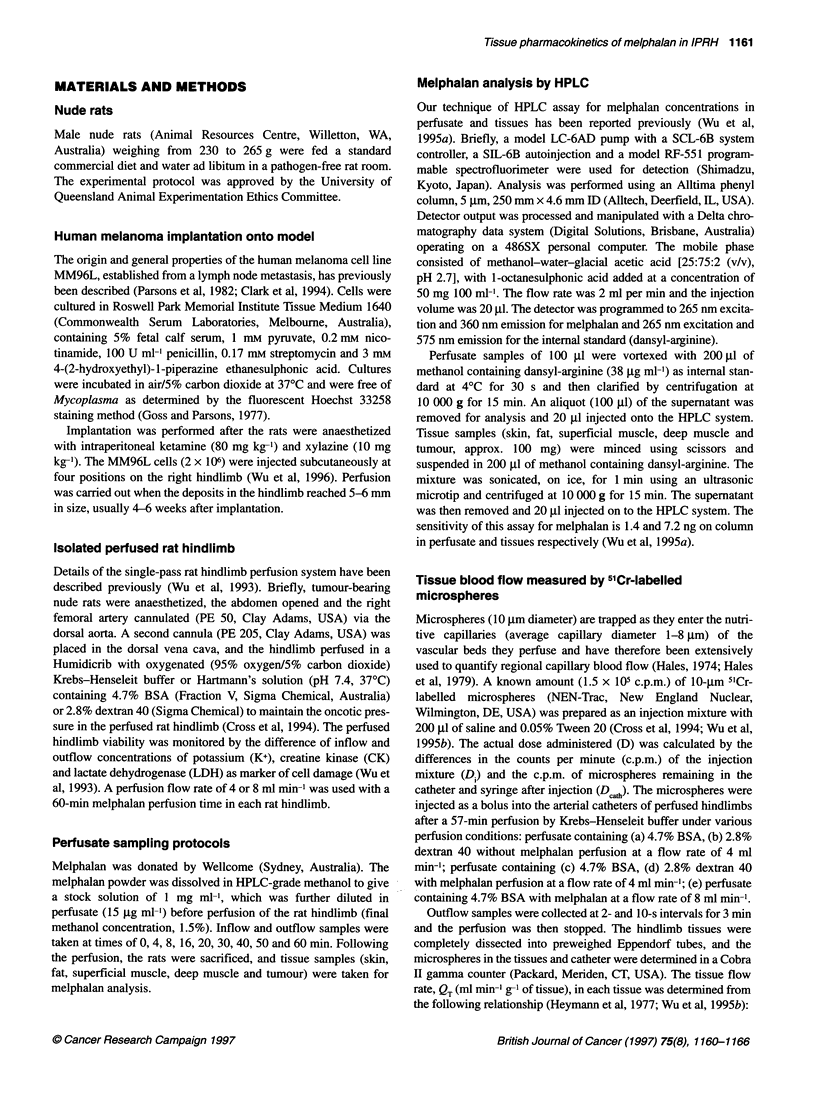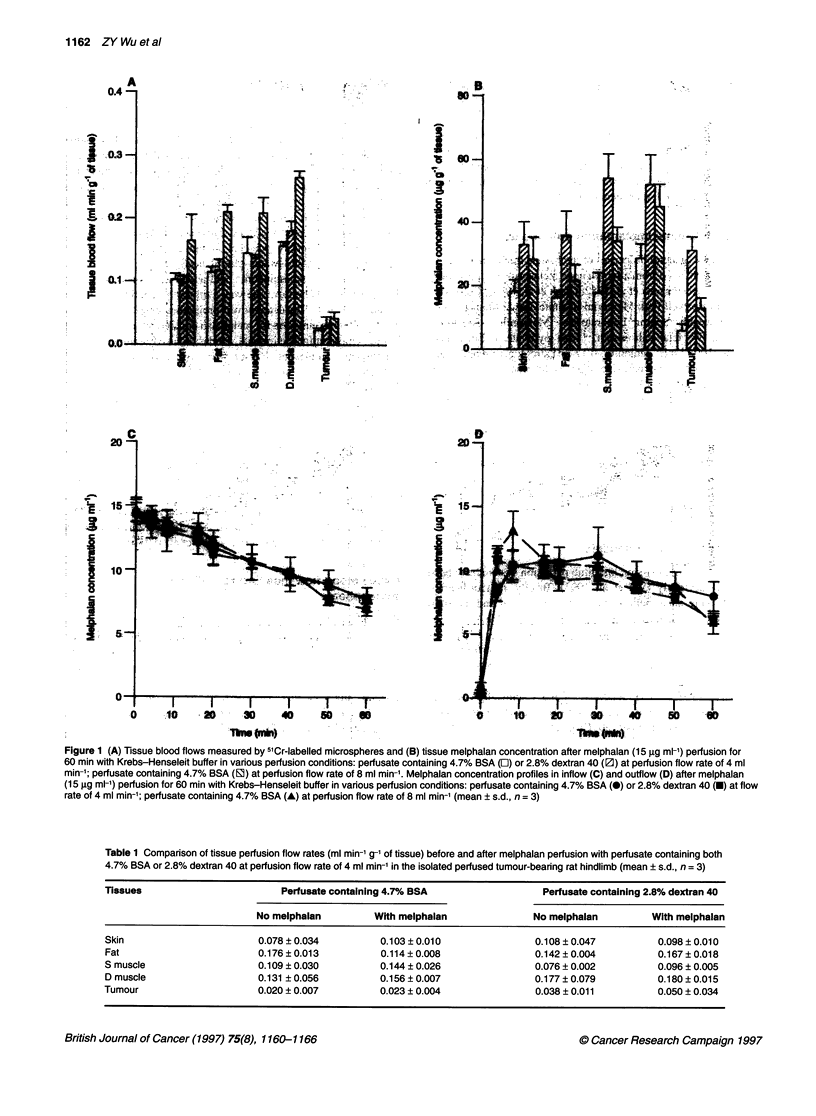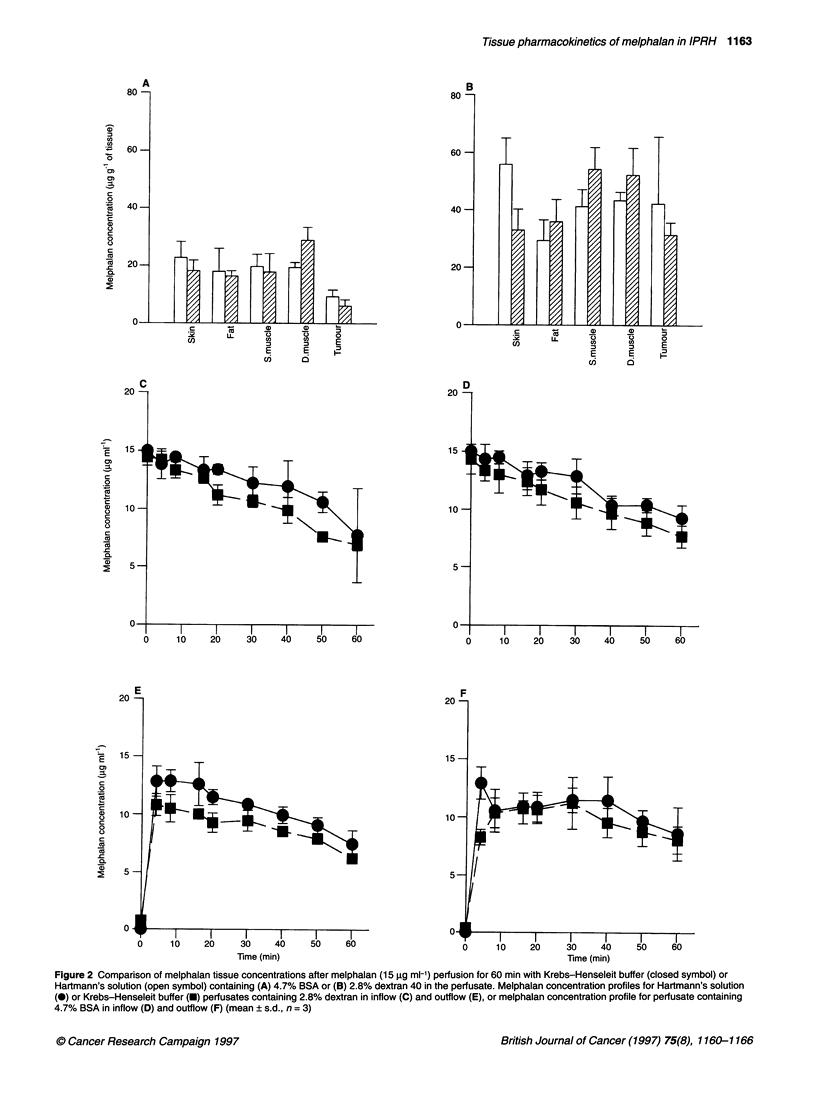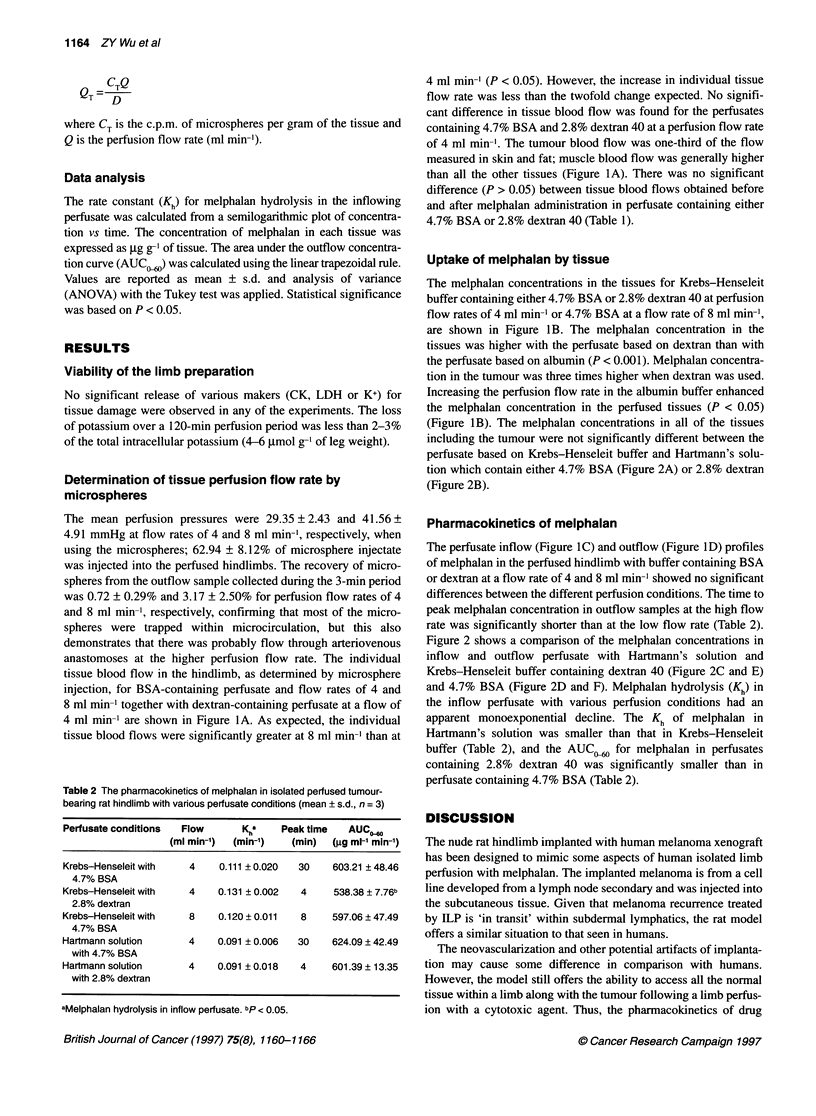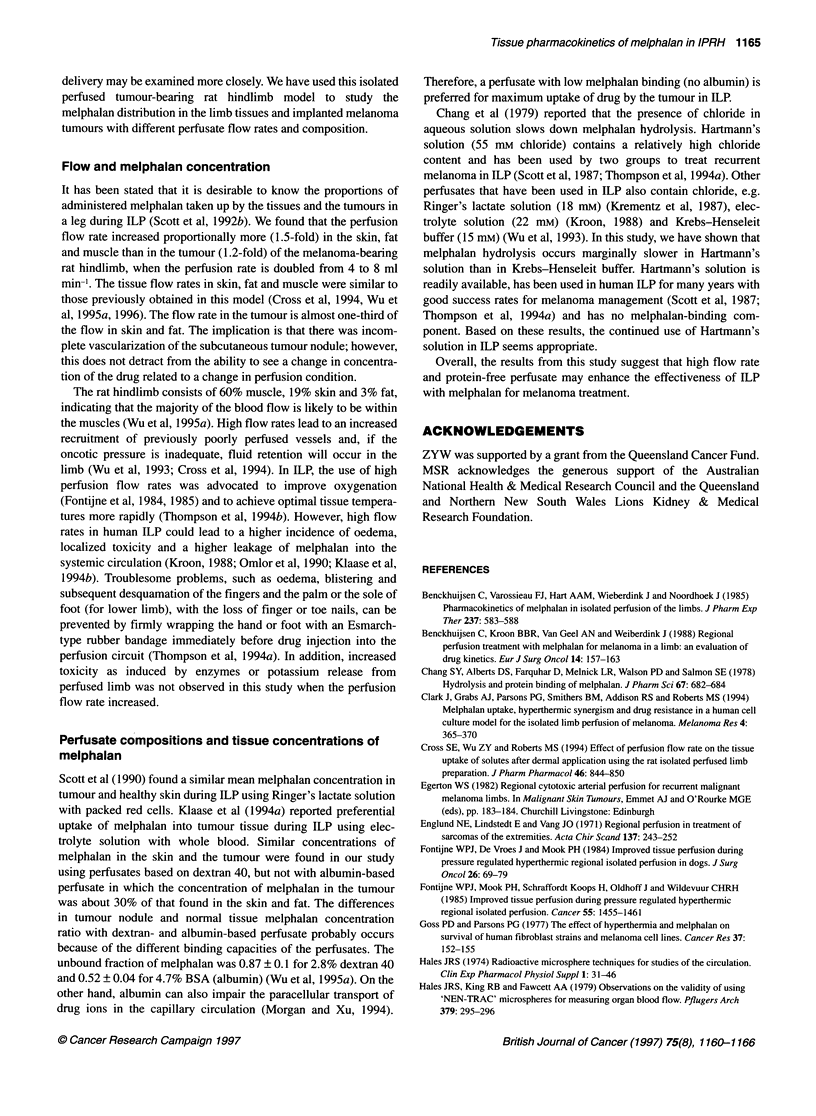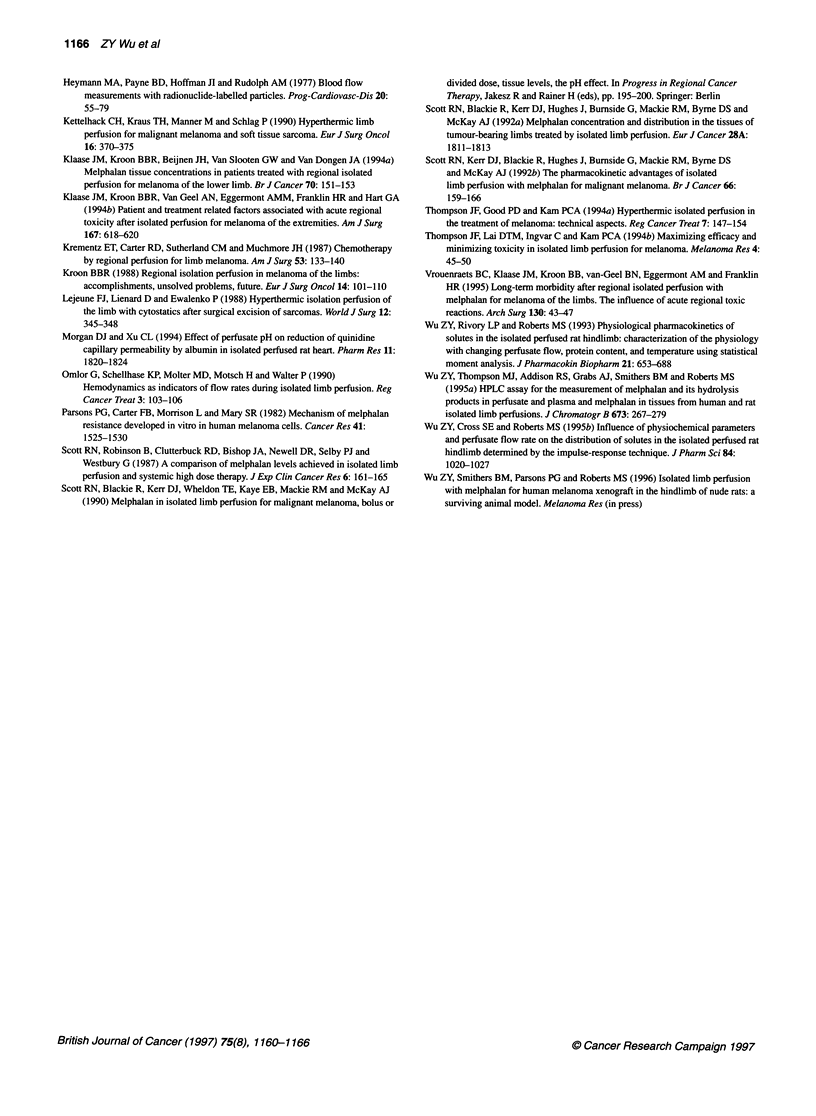# The effects of perfusion conditions on melphalan distribution in the isolated perfused rat hindlimb bearing a human melanoma xenograft.

**DOI:** 10.1038/bjc.1997.200

**Published:** 1997

**Authors:** Z. Y. Wu, B. M. Smithers, P. G. Parsons, M. S. Roberts

**Affiliations:** Department of Medicine, University of Queensland, Princess Alexandra Hospital, Brisbane, Australia.

## Abstract

An isolated rat hindlimb perfusion model carrying xenografts of the human melanoma cell line MM96 was used to study the effects of perfusion conditions on melphalan distribution. Krebs-Henseleit buffer and Hartmann's solution containing 4.7% bovine serum albumin (BSA) or 2.8% dextran 40 were used as perfusates. Melphalan concentrations in perfusate, tumour nodules and normal tissues were measured using high-performance liquid chromatography (HPLC). Increasing the perfusion flow rates (from 4 to 8 ml min(-1)) resulted in higher tissue blood flow (determined with 51Cr-labelled microspheres) and melphalan uptake by tumour and normal tissues. The distribution of melphalan within tumour nodules and normal tissues was similar for both Krebs-Henseleit buffer and Hartmann's solution; however, tissue concentrations of melphalan were significantly higher for a perfusate containing 2.8% dextran 40 than for one containing 4.7% BSA. The melphalan concentration in the tumour was one-third of that found in the skin if the perfusate contained 4.7% BSA. In conclusion, this study has shown that a high perfusion flow enhances the delivery of melphalan into implanted tumour nodules and normal tissues, and a perfusate with low melphalan binding (no albumin) is preferred for maximum uptake of drug by the tumour.


British Journal of Cancer (1997) 75(8), 1160–1166
© 1997 Cancer Research Campaign

### The effects of perfusion conditions on melphalan distribution in the isolated perfused rat hindlimb bearing a human melanoma xenograft

**ZY Wu[1,2], BM Smithers[2], PG Parsons[3] and MS Roberts[1]**

Departments of [1]Medicine and [2]Surgery, University of Queensland, Princess Alexandra Hospital, Brisbane, Qld 4102, Australia; [3]Queensland Cancer Fund Laboratories, Queensland Institute of Medical Research, Herston, Qld 4029, Australia

**Summary** An isolated rat hindlimb perfusion model carrying xenografts of the human melanoma cell line MM96 was used to study the effects of perfusion conditions on melphalan distribution. Krebs–Henseleit buffer and Hartmann's solution containing 4.7% bovine serum albumin (BSA) or 2.8% dextran 40 were used as perfusates. Melphalan concentrations in perfusate, tumour nodules and normal tissues were measured using high-performance liquid chromatography (HPLC). Increasing the perfusion flow rates (from 4 to 8 ml min$^{-1}$) resulted in higher tissue blood flow (determined with $^{51}$Cr-labelled microspheres) and melphalan uptake by tumour and normal tissues. The distribution of melphalan within tumour nodules and normal tissues was similar for both Krebs–Henseleit buffer and Hartmann's solution; however, tissue concentrations of melphalan were significantly higher for a perfusate containing 2.8% dextran 40 than for one containing 4.7% BSA. The melphalan concentration in the tumour was one-third of that found in the skin if the perfusate contained 4.7% BSA. In conclusion, this study has shown that a high perfusion flow enhances the delivery of melphalan into implanted tumour nodules and normal tissues, and a perfusate with low melphalan binding (no albumin) is preferred for maximum uptake of drug by the tumour.

**Keywords**: melphalan; tissue distribution; perfusion flow; isolated perfused tumour-bearing rat hindlimb; protein binding

In the management of malignant melanoma, isolated limb perfusion (ILP) with melphalan is an effective method of control for loco-regional advanced disease and may be an effective adjuvant to surgery in the treatment of high-risk primary lesions (Kroon, 1988). To assess the amount of cytostatic drug taken up by the tissues, pharmacokinetic studies have usually been based on the area under the concentration – time curve of melphalan in the perfusate (Benckhuijsen et al, 1985, 1988). Of more direct clinical relevance is the targeting of melphalan within melanoma nodules relative to normal tissues. Currently, there are limited data on melphalan distribution in melanoma nodules and within the tissues of the tumour-bearing limb during ILP (Scott et al, 1990; Klaase et al, 1994*a*).

Although ILP has been in use for many years to treat recurrent melanoma restricted to the limbs (Krementz et al, 1987; Scott et al, 1992*a*; Thompson et al, 1994*a*) and sarcomas of the extremities (Englund et al, 1971; Lejeune et al, 1988; Kettelhack et al, 1990), the optimal perfusion conditions have been ill defined. Currently, different perfusate compositions are used in the different centres undertaking ILP: Ringer's lactate solution with packed red cells or whole blood (Krementz et al, 1987; Scott et al, 1992*a*); electrolyte solution with whole blood (Kroon, 1988; Klasse et al, 1994*a*); or Hartmann's solution (high chloride content) with packed red cells (Thompson et al, 1994*a*) and the inclusion of dextran to maintain oncotic pressure (Egerton, 1982). We are not aware of any study that has compared the effects of variations in perfusate composition on melphalan distribution within tumour nodules and normal tissues during ILP.

Perfusion flow rate may affect both the physiology of the limb and the uptake of melphalan into tumour nodules. An argument against the use of high perfusion flow rates in ILP is the potential increased incidence of oedema, localized toxicity and leakage of melphalan to the systemic circulation with higher morbidity (Kroon, 1988; Omlor et al, 1990; Klaase et al, 1994*b*; Vrouenraets et al, 1995). However, flow rates higher than the physiological flow have been advocated to provide higher oxygenation (Fontijne et al, 1984, 1985) or enable optimal tissue temperatures to be achieved more rapidly (Thompson et al, 1994*a*). We have found that high flow rates also lead to an increased recruitment of vessels and, if the oncotic pressure is inadequate, fluid retention in limbs (Wu et al, 1993; Cross et al, 1994). Thus, one needs to feel confident that an increase in flow rate will improve tumour exposure to the melphalan.

An isolated perfused tumour-bearing rat hindlimb (Wu et al, 1993, 1996) was used to study the extent to which melphalan targeted the tumour nodules. The aims of the present study were: (1) to compare melphalan distribution in melanoma nodules within the tissues in the isolated perfused rat hindlimb using the following perfusate compositions: (a) Krebs–Henseleit buffer containing 4.7% bovine serum albumin (BSA), (b) Krebs–Henseleit buffer containing 2.8% dextran 40, (c) Hartmann's solution containing 4.7% BSA and (d) Hartmann's solution containing 2.8% dextran 40; and (2) to compare melphalan uptake into tumour and normal tissues with perfusion flow rates of 4 and 8 ml min$^{-1}$ by using Krebs–Henseleit buffer.

*Received 29 August 1996*
*Revised 22 October 1996*
*Accepted 25 October 1996*

*Correspondence to*: MS Roberts

### MATERIALS AND METHODS

### Nude rats

Male nude rats (Animal Resources Centre, Willetton, WA, Australia) weighing from 230 to 265 g were fed a standard commercial diet and water ad libitum in a pathogen-free rat room. The experimental protocol was approved by the University of Queensland Animal Experimentation Ethics Committee.

### Human melanoma implantation onto model

The origin and general properties of the human melanoma cell line MM96L, established from a lymph node metastasis, has previously been described (Parsons et al, 1982; Clark et al, 1994). Cells were cultured in Roswell Park Memorial Institute Tissue Medium 1640 (Commonwealth Serum Laboratories, Melbourne, Australia), containing 5% fetal calf serum, 1 mM pyruvate, 0.2 mM nicotinamide, 100 U ml$^{-1}$ penicillin, 0.17 mM streptomycin and 3 mM 4-(2-hydroxyethyl)-1-piperazine ethanesulphonic acid. Cultures were incubated in air/5% carbon dioxide at 37°C and were free of *Mycoplasma* as determined by the fluorescent Hoechst 33258 staining method (Goss and Parsons, 1977).

Implantation was performed after the rats were anaesthetized with intraperitoneal ketamine (80 mg kg$^{-1}$) and xylazine (10 mg kg$^{-1}$). The MM96L cells (2 × 10$^{6}$) were injected subcutaneously at four positions on the right hindlimb (Wu et al, 1996). Perfusion was carried out when the deposits in the hindlimb reached 5–6 mm in size, usually 4–6 weeks after implantation.

### Isolated perfused rat hindlimb

Details of the single-pass rat hindlimb perfusion system have been described previously (Wu et al, 1993). Briefly, tumour-bearing nude rats were anaesthetized, the abdomen opened and the right femoral artery cannulated (PE 50, Clay Adams, USA) via the dorsal aorta. A second cannula (PE 205, Clay Adams, USA) was placed in the dorsal vena cava, and the hindlimb perfused in a Humidicrib with oxygenated (95% oxygen/5% carbon dioxide) Krebs–Henseleit buffer or Hartmann's solution (pH 7.4, 37°C) containing 4.7% BSA (Fraction V, Sigma Chemical, Australia) or 2.8% dextran 40 (Sigma Chemical) to maintain the oncotic pressure in the perfused rat hindlimb (Cross et al, 1994). The perfused hindlimb viability was monitored by the difference of inflow and outflow concentrations of potassium ($K^+$), creatine kinase (CK) and lactate dehydrogenase (LDH) as marker of cell damage (Wu et al, 1993). A perfusion flow rate of 4 or 8 ml min$^{-1}$ was used with a 60-min melphalan perfusion time in each rat hindlimb.

### Perfusate sampling protocols

Melphalan was donated by Wellcome (Sydney, Australia). The melphalan powder was dissolved in HPLC-grade methanol to give a stock solution of 1 mg ml$^{-1}$, which was further diluted in perfusate (15 µg ml$^{-1}$) before perfusion of the rat hindlimb (final methanol concentration, 1.5%). Inflow and outflow samples were taken at times of 0, 4, 8, 16, 20, 30, 40, 50 and 60 min. Following the perfusion, the rats were sacrificed, and tissue samples (skin, fat, superficial muscle, deep muscle and tumour) were taken for melphalan analysis.

### Melphalan analysis by HPLC

Our technique of HPLC assay for melphalan concentrations in perfusate and tissues has been reported previously (Wu et al, 1995*a*). Briefly, a model LC-6AD pump with a SCL-6B system controller, a SIL-6B autoinjection and a model RF-551 programmable spectrofluorimeter were used for detection (Shimadzu, Kyoto, Japan). Analysis was performed using an Alltima phenyl column, 5 µm, 250 mm × 4.6 mm ID (Alltech, Deerfield, IL, USA). Detector output was processed and manipulated with a Delta chromatography data system (Digital Solutions, Brisbane, Australia) operating on a 486SX personal computer. The mobile phase consisted of methanol–water–glacial acetic acid [25:75:2 (v/v), pH 2.7], with 1-octanesulphonic acid added at a concentration of 50 mg 100 ml$^{-1}$. The flow rate was 2 ml per min and the injection volume was 20 µl. The detector was programmed to 265 nm excitation and 360 nm emission for melphalan and 265 nm excitation and 575 nm emission for the internal standard (dansyl-arginine).

Perfusate samples of 100 µl were vortexed with 200 µl of methanol containing dansyl-arginine (38 µg ml$^{-1}$) as internal standard at 4°C for 30 s and then clarified by centrifugation at 10 000 **g** for 15 min. An aliquot (100 µl) of the supernatant was removed for analysis and 20 µl injected onto the HPLC system. Tissue samples (skin, fat, superficial muscle, deep muscle and tumour, approx. 100 mg) were minced using scissors and suspended in 200 µl of methanol containing dansyl-arginine. The mixture was sonicated, on ice, for 1 min using an ultrasonic microtip and centrifuged at 10 000 **g** for 15 min. The supernatant was then removed and 20 µl injected on to the HPLC system. The sensitivity of this assay for melphalan is 1.4 and 7.2 ng on column in perfusate and tissues respectively (Wu et al, 1995*a*).

### Tissue blood flow measured by $^{51}$Cr-labelled microspheres

Microspheres (10 µm diameter) are trapped as they enter the nutritive capillaries (average capillary diameter 1–8 µm) of the vascular beds they perfuse and have therefore been extensively used to quantify regional capillary blood flow (Hales, 1974; Hales et al, 1979). A known amount (1.5 × 10$^{5}$ c.p.m.) of 10-µm $^{51}$Cr-labelled microspheres (NEN-Trac, New England Nuclear, Wilmington, DE, USA) was prepared as an injection mixture with 200 µl of saline and 0.05% Tween 20 (Cross et al, 1994; Wu et al, 1995*b*). The actual dose administered (D) was calculated by the differences in the counts per minute (c.p.m.) of the injection mixture ($D_i$) and the c.p.m. of microspheres remaining in the catheter and syringe after injection ($D_{cath}$). The microspheres were injected as a bolus into the arterial catheters of perfused hindlimbs after a 57-min perfusion by Krebs–Henseleit buffer under various perfusion conditions: perfusate containing (a) 4.7% BSA, (b) 2.8% dextran 40 without melphalan perfusion at a flow rate of 4 ml min$^{-1}$; perfusate containing (c) 4.7% BSA, (d) 2.8% dextran 40 with melphalan perfusion at a flow rate of 4 ml min$^{-1}$; (e) perfusate containing 4.7% BSA with melphalan at a flow rate of 8 ml min$^{-1}$.

Outflow samples were collected at 2- and 10-s intervals for 3 min and the perfusion was then stopped. The hindlimb tissues were completely dissected into preweighed Eppendorf tubes, and the microspheres in the tissues and catheter were determined in a Cobra II gamma counter (Packard, Meriden, CT, USA). The tissue flow rate, $Q_T$ (ml min$^{-1}$ g$^{-1}$ of tissue), in each tissue was determined from the following relationship (Heymann et al, 1977; Wu et al, 1995*b*):

© Cancer Research Campaign 1997

**Figure 1** (**A**) Tissue blood flows measured by $^{51}$Cr-labelled microspheres and (**B**) tissue melphalan concentration after melphalan (15 μg ml$^{-1}$) perfusion for 60 min with Krebs–Henseleit buffer in various perfusion conditions: perfusate containing 4.7% BSA (□) or 2.8% dextran 40 (▨) at perfusion flow rate of 4 ml min$^{-1}$; perfusate containing 4.7% BSA (▧) at perfusion flow rate of 8 ml min$^{-1}$. Melphalan concentration profiles in inflow (**C**) and outflow (**D**) after melphalan (15 μg ml$^{-1}$) perfusion for 60 min with Krebs–Henseleit buffer in various perfusion conditions: perfusate containing 4.7% BSA (●) or 2.8% dextran 40 (■) at flow rate of 4 ml min$^{-1}$; perfusate containing 4.7% BSA (▲) at perfusion flow rate of 8 ml min$^{-1}$ (mean ± s.d., *n* = 3)

**Table 1** Comparison of tissue perfusion flow rates (ml min$^{-1}$ g$^{-1}$ of tissue) before and after melphalan perfusion with perfusate containing both 4.7% BSA or 2.8% dextran 40 at perfusion flow rate of 4 ml min$^{-1}$ in the isolated perfused tumour-bearing rat hindlimb (mean ± s.d., *n* = 3)

| Tissues | Perfusate containing 4.7% BSA | | Perfusate containing 2.8% dextran 40 | |
|---|---|---|---|---|
| | No melphalan | With melphalan | No melphalan | With melphalan |
| Skin | 0.078 ± 0.034 | 0.103 ± 0.010 | 0.108 ± 0.047 | 0.098 ± 0.010 |
| Fat | 0.176 ± 0.013 | 0.114 ± 0.008 | 0.142 ± 0.004 | 0.167 ± 0.018 |
| S muscle | 0.109 ± 0.030 | 0.144 ± 0.026 | 0.076 ± 0.002 | 0.096 ± 0.005 |
| D muscle | 0.131 ± 0.056 | 0.156 ± 0.007 | 0.177 ± 0.079 | 0.180 ± 0.015 |
| Tumour | 0.020 ± 0.007 | 0.023 ± 0.004 | 0.038 ± 0.011 | 0.050 ± 0.034 |

© Cancer Research Campaign 1997

**Figure 2** Comparison of melphalan tissue concentrations after melphalan (15 μg $ml^{-1}$) perfusion for 60 min with Krebs–Henseleit buffer (closed symbol) or Hartmann's solution (open symbol) containing (**A**) 4.7% BSA or (**B**) 2.8% dextran 40 in the perfusate. Melphalan concentration profiles for Hartmann's solution (●) or Krebs–Henseleit buffer (■) perfusates containing 2.8% dextran in inflow (**C**) and outflow (**E**), or melphalan concentration profile for perfusate containing 4.7% BSA in inflow (**D**) and outflow (**F**) (mean ± s.d., *n* = 3)

© *Cancer Research Campaign 1997*

$$Q_T = \frac{C_T Q}{D}$$

where $C_T$ is the c.p.m. of microspheres per gram of the tissue and $Q$ is the perfusion flow rate (ml $min^{-1}$).

### Data analysis

The rate constant ($K_h$) for melphalan hydrolysis in the inflowing perfusate was calculated from a semilogarithmic plot of concentration *vs* time. The concentration of melphalan in each tissue was expressed as µg $g^{-1}$ of tissue. The area under the outflow concentration curve ($AUC_{0-60}$) was calculated using the linear trapezoidal rule. Values are reported as mean ± s.d. and analysis of variance (ANOVA) with the Tukey test was applied. Statistical significance was based on $P < 0.05$.

### RESULTS

### Viability of the limb preparation

No significant release of various makers (CK, LDH or $K^+$) for tissue damage were observed in any of the experiments. The loss of potassium over a 120-min perfusion period was less than 2–3% of the total intracellular potassium (4–6 µmol $g^{-1}$ of leg weight).

### Determination of tissue perfusion flow rate by microspheres

The mean perfusion pressures were 29.35 ± 2.43 and 41.56 ± 4.91 mmHg at flow rates of 4 and 8 ml $min^{-1}$, respectively, when using the microspheres; 62.94 ± 8.12% of microsphere injectate was injected into the perfused hindlimbs. The recovery of microspheres from the outflow sample collected during the 3-min period was 0.72 ± 0.29% and 3.17 ± 2.50% for perfusion flow rates of 4 and 8 ml $min^{-1}$, respectively, confirming that most of the microspheres were trapped within microcirculation, but this also demonstrates that there was probably flow through arteriovenous anastomoses at the higher perfusion flow rate. The individual tissue blood flow in the hindlimb, as determined by microsphere injection, for BSA-containing perfusate and flow rates of 4 and 8 ml $min^{-1}$ together with dextran-containing perfusate at a flow of 4 ml $min^{-1}$ are shown in Figure 1A. As expected, the individual tissue blood flows were significantly greater at 8 ml $min^{-1}$ than at 4 ml $min^{-1}$ ($P < 0.05$). However, the increase in individual tissue flow rate was less than the twofold change expected. No significant difference in tissue blood flow was found for the perfusates containing 4.7% BSA and 2.8% dextran 40 at a perfusion flow rate of 4 ml $min^{-1}$. The tumour blood flow was one-third of the flow measured in skin and fat; muscle blood flow was generally higher than all the other tissues (Figure 1A). There was no significant difference ($P > 0.05$) between tissue blood flows obtained before and after melphalan administration in perfusate containing either 4.7% BSA or 2.8% dextran 40 (Table 1).

**Table 2** The pharmacokinetics of melphalan in isolated perfused tumour-bearing rat hindlimb with various perfusate conditions (mean ± s.d., $n = 3$)

| Perfusate conditions | Flow (ml $min^{-1}$) | $K_h$[a] ($min^{-1}$) | Peak time (min) | $AUC_{0-60}$ (µg $ml^{-1}$ $min^{-1}$) |
|---|---|---|---|---|
| Krebs–Henseleit with 4.7% BSA | 4 | 0.111 ± 0.020 | 30 | 603.21 ± 48.46 |
| Krebs–Henseleit with 2.8% dextran | 4 | 0.131 ± 0.002 | 4 | 538.38 ± 7.76[b] |
| Krebs–Henseleit with 4.7% BSA | 8 | 0.120 ± 0.011 | 8 | 597.06 ± 47.49 |
| Hartmann solution with 4.7% BSA | 4 | 0.091 ± 0.006 | 30 | 624.09 ± 42.49 |
| Hartmann solution with 2.8% dextran | 4 | 0.091 ± 0.018 | 4 | 601.39 ± 13.35 |

[a]Melphalan hydrolysis in inflow perfusate. [b]$P < 0.05$.

### Uptake of melphalan by tissue

The melphalan concentrations in the tissues for Krebs–Henseleit buffer containing either 4.7% BSA or 2.8% dextran 40 at perfusion flow rates of 4 ml $min^{-1}$ or 4.7% BSA at a flow rate of 8 ml $min^{-1}$, are shown in Figure 1B. The melphalan concentration in the tissues was higher with the perfusate based on dextran than with the perfusate based on albumin ($P < 0.001$). Melphalan concentration in the tumour was three times higher when dextran was used. Increasing the perfusion flow rate in the albumin buffer enhanced the melphalan concentration in the perfused tissues ($P < 0.05$) (Figure 1B). The melphalan concentrations in all of the tissues including the tumour were not significantly different between the perfusate based on Krebs–Henseleit buffer and Hartmann's solution which contain either 4.7% BSA (Figure 2A) or 2.8% dextran (Figure 2B).

### Pharmacokinetics of melphalan

The perfusate inflow (Figure 1C) and outflow (Figure 1D) profiles of melphalan in the perfused hindlimb with buffer containing BSA or dextran at a flow rate of 4 and 8 ml $min^{-1}$ showed no significant differences between the different perfusion conditions. The time to peak melphalan concentration in outflow samples at the high flow rate was significantly shorter than at the low flow rate (Table 2). Figure 2 shows a comparison of the melphalan concentrations in inflow and outflow perfusate with Hartmann's solution and Krebs–Henseleit buffer containing dextran 40 (Figure 2C and E) and 4.7% BSA (Figure 2D and F). Melphalan hydrolysis ($K_h$) in the inflow perfusate with various perfusion conditions had an apparent monoexponential decline. The $K_h$ of melphalan in Hartmann's solution was smaller than that in Krebs–Henseleit buffer (Table 2), and the $AUC_{0-60}$ for melphalan in perfusates containing 2.8% dextran 40 was significantly smaller than in perfusate containing 4.7% BSA (Table 2).

### DISCUSSION

The nude rat hindlimb implanted with human melanoma xenograft has been designed to mimic some aspects of human isolated limb perfusion with melphalan. The implanted melanoma is from a cell line developed from a lymph node secondary and was injected into the subcutaneous tissue. Given that melanoma recurrence treated by ILP is 'in transit' within subdermal lymphatics, the rat model offers a similar situation to that seen in humans.

The neovascularization and other potential artifacts of implantation may cause some difference in comparison with humans. However, the model still offers the ability to access all the normal tissue within a limb along with the tumour following a limb perfusion with a cytotoxic agent. Thus, the pharmacokinetics of drug

© Cancer Research Campaign 1997

delivery may be examined more closely. We have used this isolated perfused tumour-bearing rat hindlimb model to study the melphalan distribution in the limb tissues and implanted melanoma tumours with different perfusate flow rates and composition.

### Flow and melphalan concentration

It has been stated that it is desirable to know the proportions of administered melphalan taken up by the tissues and the tumours in a leg during ILP (Scott et al, 1992*b*). We found that the perfusion flow rate increased proportionally more (1.5-fold) in the skin, fat and muscle than in the tumour (1.2-fold) of the melanoma-bearing rat hindlimb, when the perfusion rate is doubled from 4 to 8 ml $min^{-1}$. The tissue flow rates in skin, fat and muscle were similar to those previously obtained in this model (Cross et al, 1994, Wu et al, 1995*a*, 1996). The flow rate in the tumour is almost one-third of the flow in skin and fat. The implication is that there was incomplete vascularization of the subcutaneous tumour nodule; however, this does not detract from the ability to see a change in concentration of the drug related to a change in perfusion condition.

The rat hindlimb consists of 60% muscle, 19% skin and 3% fat, indicating that the majority of the blood flow is likely to be within the muscles (Wu et al, 1995*a*). High flow rates lead to an increased recruitment of previously poorly perfused vessels and, if the oncotic pressure is inadequate, fluid retention will occur in the limb (Wu et al, 1993; Cross et al, 1994). In ILP, the use of high perfusion flow rates was advocated to improve oxygenation (Fontijne et al, 1984, 1985) and to achieve optimal tissue temperatures more rapidly (Thompson et al, 1994*b*). However, high flow rates in human ILP could lead to a higher incidence of oedema, localized toxicity and a higher leakage of melphalan into the systemic circulation (Kroon, 1988; Omlor et al, 1990; Klaase et al, 1994*b*). Troublesome problems, such as oedema, blistering and subsequent desquamation of the fingers and the palm or the sole of foot (for lower limb), with the loss of finger or toe nails, can be prevented by firmly wrapping the hand or foot with an Esmarch-type rubber bandage immediately before drug injection into the perfusion circuit (Thompson et al, 1994*a*). In addition, increased toxicity as induced by enzymes or potassium release from perfused limb was not observed in this study when the perfusion flow rate increased.

### Perfusate compositions and tissue concentrations of melphalan

Scott et al (1990) found a similar mean melphalan concentration in tumour and healthy skin during ILP using Ringer's lactate solution with packed red cells. Klaase et al (1994*a*) reported preferential uptake of melphalan into tumour tissue during ILP using electrolyte solution with whole blood. Similar concentrations of melphalan in the skin and the tumour were found in our study using perfusates based on dextran 40, but not with albumin-based perfusate in which the concentration of melphalan in the tumour was about 30% of that found in the skin and fat. The differences in tumour nodule and normal tissue melphalan concentration ratio with dextran- and albumin-based perfusate probably occurs because of the different binding capacities of the perfusates. The unbound fraction of melphalan was $0.87 \pm 0.1$ for 2.8% dextran 40 and $0.52 \pm 0.04$ for 4.7% BSA (albumin) (Wu et al, 1995*a*). On the other hand, albumin can also impair the paracellular transport of drug ions in the capillary circulation (Morgan and Xu, 1994). Therefore, a perfusate with low melphalan binding (no albumin) is preferred for maximum uptake of drug by the tumour in ILP.

Chang et al (1979) reported that the presence of chloride in aqueous solution slows down melphalan hydrolysis. Hartmann's solution (55 mM chloride) contains a relatively high chloride content and has been used by two groups to treat recurrent melanoma in ILP (Scott et al, 1987; Thompson et al, 1994*a*). Other perfusates that have been used in ILP also contain chloride, e.g. Ringer's lactate solution (18 mM) (Krementz et al, 1987), electrolyte solution (22 mM) (Kroon, 1988) and Krebs–Henseleit buffer (15 mM) (Wu et al, 1993). In this study, we have shown that melphalan hydrolysis occurs marginally slower in Hartmann's solution than in Krebs–Henseleit buffer. Hartmann's solution is readily available, has been used in human ILP for many years with good success rates for melanoma management (Scott et al, 1987; Thompson et al, 1994*a*) and has no melphalan-binding component. Based on these results, the continued use of Hartmann's solution in ILP seems appropriate.

Overall, the results from this study suggest that high flow rate and protein-free perfusate may enhance the effectiveness of ILP with melphalan for melanoma treatment.

### ACKNOWLEDGEMENTS

ZYW was supported by a grant from the Queensland Cancer Fund. MSR acknowledges the generous support of the Australian National Health & Medical Research Council and the Queensland and Northern New South Wales Lions Kidney & Medical Research Foundation.

### REFERENCES

Benckhuijsen C, Varossieau FJ, Hart AAM, Wieberdink J and Noordhoek J (1985) Pharmacokinetics of melphalan in isolated perfusion of the limbs. *J Pharm Exp Ther* **237**: 583–588

Benckhuijsen C, Kroon BBR, Van Geel AN and Weiberdink J (1988) Regional perfusion treatment with melphalan for melanoma in a limb: an evaluation of drug kinetics. *Eur J Surg Oncol* **14**: 157–163

Chang SY, Alberts DS, Farquhar D, Melnick LR, Walson PD and Salmon SE (1978) Hydrolysis and protein binding of melphalan. *J Pharm Sci* **67**: 682–684

Clark J, Grabs AJ, Parsons PG, Smithers BM, Addison RS and Roberts MS (1994) Melphalan uptake, hyperthermic synergism and drug resistance in a human cell culture model for the isolated limb perfusion of melanoma. *Melanoma Res* **4**: 365–370

Cross SE, Wu ZY and Roberts MS (1994) Effect of perfusion flow rate on the tissue uptake of solutes after dermal application using the rat isolated perfused limb preparation. *J Pharm Pharmacol* **46**: 844–850

Egerton WS (1982) Regional cytotoxic arterial perfusion for recurrent malignant melanoma limbs. In *Malignant Skin Tumours*, Emmet AJ and O'Rourke MGE (eds), pp. 183–184. Churchill Livingstone: Edinburgh

Englund NE, Lindstedt E and Vang JO (1971) Regional perfusion in treatment of sarcomas of the extremities. *Acta Chir Scand* **137**: 243–252

Fontijne WPJ, De Vroes J and Mook PH (1984) Improved tissue perfusion during pressure regulated hyperthermic regional isolated perfusion in dogs. *J Surg Oncol* **26**: 69–79

Fontijne WPJ, Mook PH, Schraffordt Koops H, Oldhoff J and Wildevuur CHRH (1985) Improved tissue perfusion during pressure regulated hyperthermic regional isolated perfusion. *Cancer* **55**: 1455–1461

Goss PD and Parsons PG (1977) The effect of hyperthermia and melphalan on survival of human fibroblast strains and melanoma cell lines. *Cancer Res* **37**: 152–155

Hales JRS (1974) Radioactive microsphere techniques for studies of the circulation. *Clin Exp Pharmacol Physiol Suppl* **1**: 31–46

Hales JRS, King RB and Fawcett AA (1979) Observations on the validity of using 'NEN-TRAC' microspheres for measuring organ blood flow. *Pflugers Arch* **379**: 295–296

© Cancer Research Campaign 1997 

Heymann MA, Payne BD, Hoffman JI and Rudolph AM (1977) Blood flow measurements with radionuclide-labelled particles. *Prog-Cardiovasc-Dis* **20**: 55–79

Kettelhack CH, Kraus TH, Manner M and Schlag P (1990) Hyperthermic limb perfusion for malignant melanoma and soft tissue sarcoma. *Eur J Surg Oncol* **16**: 370–375

Klaase JM, Kroon BBR, Beijnen JH, Van Slooten GW and Van Dongen JA (1994*a*) Melphalan tissue concentrations in patients treated with regional isolated perfusion for melanoma of the lower limb. *Br J Cancer* **70**: 151–153

Klaase JM, Kroon BBR, Van Geel AN, Eggermont AMM, Franklin HR and Hart GA (1994*b*) Patient and treatment related factors associated with acute regional toxicity after isolated perfusion for melanoma of the extremities. *Am J Surg* **167**: 618–620

Krementz ET, Carter RD, Sutherland CM and Muchmore JH (1987) Chemotherapy by regional perfusion for limb melanoma. *Am J Surg* **53**: 133–140

Kroon BBR (1988) Regional isolation perfusion in melanoma of the limbs: accomplishments, unsolved problems, future. *Eur J Surg Oncol* **14**: 101–110

Lejeune FJ, Lienard D and Ewalenko P (1988) Hyperthermic isolation perfusion of the limb with cytostatics after surgical excision of sarcomas. *World J Surg* **12**: 345–348

Morgan DJ and Xu CL (1994) Effect of perfusate pH on reduction of quinidine capillary permeability by albumin in isolated perfused rat heart. *Pharm Res* **11**: 1820–1824

Omlor G, Schellhase KP, Molter MD, Motsch H and Walter P (1990) Hemodynamics as indicators of flow rates during isolated limb perfusion. *Reg Cancer Treat* **3**: 103–106

Parsons PG, Carter FB, Morrison L and Mary SR (1982) Mechanism of melphalan resistance developed in vitro in human melanoma cells. *Cancer Res* **41**: 1525–1530

Scott RN, Robinson B, Clutterbuck RD, Bishop JA, Newell DR, Selby PJ and Westbury G (1987) A comparison of melphalan levels achieved in isolated limb perfusion and systemic high dose therapy. *J Exp Clin Cancer Res* **6**: 161–165

Scott RN, Blackie R, Kerr DJ, Wheldon TE, Kaye EB, Mackie RM and McKay AJ (1990) Melphalan in isolated limb perfusion for malignant melanoma, bolus or divided dose, tissue levels, the pH effect. In *Progress in Regional Cancer Therapy*, Jakesz R and Rainer H (eds), pp. 195–200. Springer: Berlin

Scott RN, Blackie R, Kerr DJ, Hughes J, Burnside G, Mackie RM, Byrne DS and McKay AJ (1992*a*) Melphalan concentration and distribution in the tissues of tumour-bearing limbs treated by isolated limb perfusion. *Eur J Cancer* **28A**: 1811–1813

Scott RN, Kerr DJ, Blackie R, Hughes J, Burnside G, Mackie RM, Byrne DS and McKay AJ (1992*b*) The pharmacokinetic advantages of isolated limb perfusion with melphalan for malignant melanoma. *Br J Cancer* **66**: 159–166

Thompson JF, Good PD and Kam PCA (1994*a*) Hyperthermic isolated perfusion in the treatment of melanoma: technical aspects. *Reg Cancer Treat* **7**: 147–154

Thompson JF, Lai DTM, Ingvar C and Kam PCA (1994*b*) Maximizing efficacy and minimizing toxicity in isolated limb perfusion for melanoma. *Melanoma Res* **4**: 45–50

Vrouenraets BC, Klaase JM, Kroon BB, van-Geel BN, Eggermont AM and Franklin HR (1995) Long-term morbidity after regional isolated perfusion with melphalan for melanoma of the limbs. The influence of acute regional toxic reactions. *Arch Surg* **130**: 43–47

Wu ZY, Rivory LP and Roberts MS (1993) Physiological pharmacokinetics of solutes in the isolated perfused rat hindlimb: characterization of the physiology with changing perfusate flow, protein content, and temperature using statistical moment analysis. *J Pharmacokin Biopharm* **21**: 653–688

Wu ZY, Thompson MJ, Addison RS, Grabs AJ, Smithers BM and Roberts MS (1995*a*) HPLC assay for the measurement of melphalan and its hydrolysis products in perfusate and plasma and melphalan in tissues from human and rat isolated limb perfusions. *J Chromatogr B* **673**: 267–279

Wu ZY, Cross SE and Roberts MS (1995*b*) Influence of physiochemical parameters and perfusate flow rate on the distribution of solutes in the isolated perfused rat hindlimb determined by the impulse-response technique. *J Pharm Sci* **84**: 1020–1027

Wu ZY, Smithers BM, Parsons PG and Roberts MS (1996) Isolated limb perfusion with melphalan for human melanoma xenograft in the hindlimb of nude rats: a surviving animal model. *Melanoma Res* (in press)

© Cancer Research Campaign 1997